# Participatory development of an mHealth intervention delivered in general practice to increase physical activity and reduce sedentary behaviour of patients with prediabetes and type 2 diabetes (ENERGISED)

**DOI:** 10.1186/s12889-024-18384-2

**Published:** 2024-03-31

**Authors:** Jan Novak, Katerina Jurkova, Anna Lojkaskova, Andrea Jaklova, Jitka Kuhnova, Marketa Pfeiferova, Norbert Kral, Michael Janek, Dan Omcirk, Katerina Malisova, Iris Maes, Delfien Van Dyck, Charlotte Wahlich, Michael Ussher, Steriani Elavsky, Richard Cimler, Jana Pelclova, James J. Tufano, Michal Steffl, Bohumil Seifert, Tom Yates, Tess Harris, Tomas Vetrovsky

**Affiliations:** 1 Faculty of Physical Education and Sport, Charles University, Prague, Czech Republic; 2 Faculty of Science, University of Hradec Kralove, Hradec Kralove, Czech Republic; 3 Institute of General Practice, 1st Faculty of Medicine, Charles University, Prague, Czech Republic; 4 Faculty of Physical Culture, Palacky University Olomouc, Olomouc, Czech Republic; 5 Department of Movement and Sports Sciences, Ghent University, Ghent, Belgium; 6 Population Health Research Institute, St George’s University of London, London, UK; 7 Institute for Social Marketing and Health, University of Stirling, Stirling, UK; 8 Department of Human Movement Studies, University of Ostrava, Ostrava, Czech Republic; 9 Diabetes Research Centre, University of Leicester, Leicester, UK; 10 National Institute for Health Research (NIHR) Leicester Biomedical Research Centre, University Hospitals of Leicester NHS Trust and the University of Leicester, Leicester, UK

**Keywords:** Primary care, Just-in-time adaptive intervention (JITAI), Self-regulation theory, Fitbit, Wearables, Phone counselling, Text messages, Participatory development, Walking, Behaviour change techniques

## Abstract

**Background:**

The escalating global prevalence of type 2 diabetes and prediabetes presents a major public health challenge. Physical activity plays a critical role in managing (pre)diabetes; however, adherence to physical activity recommendations remains low. The ENERGISED trial was designed to address these challenges by integrating mHealth tools into the routine practice of general practitioners, aiming for a significant, scalable impact in (pre)diabetes patient care through increased physical activity and reduced sedentary behaviour.

**Methods:**

The mHealth intervention for the ENERGISED trial was developed according to the mHealth development and evaluation framework, which includes the active participation of (pre)diabetes patients. This iterative process encompasses four sequential phases: (a) conceptualisation to identify key aspects of the intervention; (b) formative research including two focus groups with (pre)diabetes patients (*n* = 14) to tailor the intervention to the needs and preferences of the target population; (c) pre-testing using think-aloud patient interviews (*n* = 7) to optimise the intervention components; and (d) piloting (*n* = 10) to refine the intervention to its final form.

**Results:**

The final intervention comprises six types of text messages, each embodying different behaviour change techniques. Some of the messages, such as those providing interim reviews of the patients’ weekly step goal or feedback on their weekly performance, are delivered at fixed times of the week. Others are triggered just in time by specific physical behaviour events as detected by the Fitbit activity tracker: for example, prompts to increase walking pace are triggered after 5 min of continuous walking; and prompts to interrupt sitting following 30 min of uninterrupted sitting. For patients without a smartphone or reliable internet connection, the intervention is adapted to ensure inclusivity. Patients receive on average three to six messages per week for 12 months. During the first six months, the text messaging is supplemented with monthly phone counselling to enable personalisation of the intervention, assistance with technical issues, and enhancement of adherence.

**Conclusions:**

The participatory development of the ENERGISED mHealth intervention, incorporating just-in-time prompts, has the potential to significantly enhance the capacity of general practitioners for personalised behavioural counselling on physical activity in (pre)diabetes patients, with implications for broader applications in primary care.

**Supplementary Information:**

The online version contains supplementary material available at 10.1186/s12889-024-18384-2.

## Background

The global prevalence of type 2 diabetes and prediabetes has risen steadily, posing significant public health challenges. In 2021, the global diabetes prevalence was estimated to be 10.5%, with an additional 9.1% of adults having impaired glucose tolerance, which places them at high risk of type 2 diabetes [1, 2].

Physical activity (PA) is a cornerstone in the management of (pre)diabetes [3, 4]. Regular PA improves glycaemic control, aids in weight management, and reduces cardiovascular risk factors [5–7]. Furthermore, reducing and interrupting prolonged sitting improves markers of metabolic health [8–10]. Despite these well-documented benefits, a significant proportion of individuals with (pre)diabetes remain insufficiently active [11, 12]. For example, a recent accelerometry study from Denmark found that 63.2% and 59.5% of participants with diabetes and prediabetes, respectively, did not adhere to the WHO recommendations of weekly minutes of moderate-to-vigorous PA, compared with 49.6% of participants without (pre)diabetes [13]. Therefore, interventions that can effectively promote and sustain PA in this population are critically needed.

Mobile health (mHealth) technologies have emerged as promising tools for delivering PA interventions [14–16]. The ubiquity of smartphones and wearable devices offers a unique opportunity to provide personalised, context-sensitive, and scalable just-in-time adaptive interventions (JITAIs), which use data from wearable sensors to intervene when it is most relevant for the patient [17, 18]. Despite the potential of mHealth, its application in diabetes care faces several challenges. These include ensuring user engagement, tailoring the intervention to individual needs and preferences, and integrating the technology seamlessly into daily life [19–21]. Additionally, there is a need to address the digital divide, as not all (pre)diabetes patients may have access to or be comfortable with using advanced technologies [22]. Therefore, designing mHealth interventions that are accessible, user-friendly, and effective in promoting sustained behaviour change is essential.

Building upon the potential of mHealth technologies in diabetes care, general practitioners (GPs) within primary care emerge as crucial players in this landscape. GPs are at the forefront of managing (pre)diabetes, especially in guiding patients towards healthier behaviours, including increased PA and reduced sedentary lifestyles [23, 24]. Despite their pivotal role, GPs often encounter time constraints, limiting their capacity for extensive behavioural counselling [25–27]. Here, mHealth interventions, when delivered in primary care, offer a valuable extension of GPs’ efforts [28, 29]. These tools can enhance patient support in a time-efficient manner, aligning with the individualised care approach essential in diabetes management. This approach not only addresses some of the key challenges of mHealth, such as user engagement and personalisation, but also capitalises on the trusted patient-GP relationship to enhance the effectiveness of these interventions [30, 31]. Consequently, integrating mHealth tools into primary care practices represents a significant step towards more effective and sustainable management of (pre)diabetes.

As a practical response to these insights, the ENERGISED trial has been designed to evaluate the effectiveness of an innovative mHealth intervention in primary care for patients with (pre)diabetes, focusing on increasing PA and reducing sedentary behaviour. The rationale and study protocol for this trial has been described previously [32]. Briefly, this 12-month pragmatic, multicentre, randomised controlled trial aims to recruit 340 patients from 21 general practices, leveraging routine health check-ups for recruitment. The trial comprises a six-month lead-in phase, where the mHealth intervention is supported by human phone counselling, followed by a six-month fully automated maintenance phase. The mHealth intervention is compared against an active control group: participants in both groups receive brief PA advice from their GP, supplemented with a Fitbit activity tracker for self-monitoring. The primary outcome is the change in average ambulatory activity, measured in steps per day via a wrist-worn accelerometer.

This paper aims to describe the participatory development and piloting of the mHealth intervention and its final version to be evaluated in the ENERGISED trial, complementing the previously published trial protocol [32]. Our decision to employ a participatory approach was driven by the recognition that the success of mHealth interventions, particularly in the context of physical activity and sedentary behaviour change, hinges on their relevance and adaptability to the end-users’ daily lives and challenges [33, 34]. This approach aligns with contemporary best practices in intervention design [35], which advocate for the active involvement of potential users to ensure interventions are not only effective in theory, but also embraced and utilised in practice [36]. By involving patients with prediabetes and type 2 diabetes in the development process, we aimed to ensure that the intervention was grounded in the real-world experiences and needs of those it seeks to support [37], thereby enhancing its potential for a significant and lasting impact and scalability to a broad population of (pre)diabetes patients within primary care.

## Methods and results

The mHealth intervention was developed according to the ‘mHealth development and evaluation framework’, which includes active participation of the target audience in focus groups and interviews [38–40]. This framework encompasses four sequential phases: (a) conceptualisation, (b) formative research, (c) pre-testing, and (d) piloting.

We present a combined overview of the methods and results for each phase, providing a cohesive narrative that aligns the development process with the corresponding outcomes, rather than separating out methods and results. We then present the finalised intervention, as implemented in the ongoing ENERGISED randomised controlled trial.

### Participants

All participants involved in the intervention’s development were patients with (pre)diabetes who fulfilled the ENERGISED trial eligibility criteria (Additional file 1), recruited by collaborating GPs from their practices in Prague, Czech Republic.

The Ethics Committee of the General University Hospital in Prague (No. 49/20) provided study approval, and all participants provided informed consent.

### Phase 1: conceptualisation

#### Methods

To reach a consensus on the key conceptual aspects of the intervention, the multidisciplinary team employed an informal decision-making process. This team comprised GPs (BS, MP, NK, TH), PA researchers (DVD, JP, MS, TY)– some of whom have extensive expertise with diabetes patients (TH, TY)– as well as psychologists and behavioural scientists (MU, SE, CW), and IT experts (JK, RC). The perspectives of GPs were deemed particularly crucial, as they are the primary agents tasked with the intervention implementation in real-world settings and they have day-to-day experience of consultations addressing physical inactivity with their (pre)diabetes patients. Engaging GPs early in the intervention development process was vital for identifying and overcoming potential barriers to implementation, such as time constraints and integration into existing workflows, while leveraging facilitators like the trusted GP-patient relationship and the GPs’ unique insights into patient needs and preferences [25, 31]. This approach aligns with prior research indicating that the early involvement of key stakeholders, especially those directly impacted by the intervention’s implementation, significantly enhances the feasibility and acceptability of health interventions [41]. The four GPs involved in the conceptualisation phase represented a diverse cross-section of practice settings, including both rural (MP) and urban (BS, NK) environments, and brought a range of experiences, with years of practice varying from recently qualified (MP) to over 30 years of experience (BS, TH). This diversity ensured a broad spectrum of insights into the challenges and opportunities of implementing the intervention across different healthcare contexts. The team included both male and female GPs, with three from the Czech Republic—where the intervention is to be implemented—to ensure the intervention’s relevance to the local healthcare system. Additionally, we included a GP from the UK (TH) with additional experience of delivering physical activity trials in primary care to incorporate an external perspective. This helped to enrich the intervention’s development, with broader insights into its potential applicability and scalability beyond the initial setting.The process began with individual team members thoroughly reviewing the latest evidence in their respective fields related to physical activity, diabetes management, behaviour change theories, mHealth technologies, and interventions related to all these areas, including our prior research [40, 42–45]. Following this, a series of meetings were convened, where team members presented their findings and proposed elements for the intervention’s design. During these meetings, facilitated discussions were held to integrate the diverse perspectives of the team, whilst considering resource and time constraints. The discussions were structured around several key conceptual aspects: underpinning theory and behaviour change techniques (BCTs); mode of physical activity and intervention goals; intervention components; and the required IT solution. The outcome of this process was a document, drafted by one of the researchers (TV), which outlined the agreed-upon key conceptual aspects forming the foundation of the intervention. This document, accompanied by a rationale for each aspect, was reviewed and approved by the entire team, guiding the subsequent phases of intervention development.

#### Results

##### Theoretical underpinning and behaviour change techniques

The mHealth intervention was underpinned by the theory of self-regulation, a psychological framework that emphasises the role of self-directed processes in guiding one’s behaviour towards achieving personal goals [46]. The intervention thus incorporates a range of self-regulatory BCTs, such as self-monitoring, goal setting, and feedback [47], to which we have allocated the same numerical codes in brackets as per Michie et al. taxonomy [48].

Self-monitoring (2.3) stands as a cornerstone of self-regulation, allowing patients to track their progress and gain insights into their PA patterns. A wealth of evidence indicates that self-monitoring can significantly increase PA levels [45, 49] and reduce sedentary behaviour [21, 43]. Goal setting (1.1) and regular goal review (1.5) further complement self-monitoring by providing patients with clear, tangible targets to strive for and a framework to evaluate their progress. Goal-setting is the key component of self-regulation [50] and one of the most potent behaviour change techniques in increasing PA [16, 51]. A recent meta-analysis estimated that setting a specific goal was associated with an increase of approximately 600 steps/day [42]. Action planning (1.4) and coping planning (1.2) aid in translating these goals into daily routines, helping patients identify specific activities, times, and contexts in which they can incorporate more PA. Action planning has been identified as one of the most frequently used BCTs in the general population [51] and patients with diabetes [52]. Furthermore, combining action planning, coping planning, and self-monitoring was more effective in increasing PA and reducing sedentary behaviour than using these BCTs alone [21]. Feedback on behaviour (2.2) serves as a continuous loop of reinforcement, allowing patients to understand where they are excelling and where there’s room for improvement. In a review of mHealth interventions to influence PA and sedentary behaviour, approximately half (46%) utilised feedback on behaviour [53], which is also commonly used in interventions targeting diabetes patients [52]. Providing information about health consequences (5.1) highlights the tangible health benefits of increased PA and the health risks of sedentary behaviour. This technique aims to enhance motivation and drive behavioural change, especially in patients with chronic conditions [54, 55], including (pre)diabetes [52]. Lastly, prompts and cues (7.1) play a crucial role in nudging patients towards increased PA and reduced sedentary behaviour in real-time. While not commonly used in traditional PA interventions, prompts are massively utilised by mHealth interventions, which facilitate easy implementation of timely reminders or suggestions, often based on real-time wearable sensor data [53, 55, 56].

Collectively, these BCTs form the backbone of our intervention, each contributing uniquely to fostering a sustained increase in PA and a decrease in sedentary behaviour among our target population.

##### Mode of physical activity and intervention goals

We identified walking as the primary mode of PA for the intervention due to its accessibility, low cost, established benefits for metabolic health [57, 58], and safety [59]. This choice is grounded in the understanding that walking can be seamlessly integrated into daily routines, making it a sustainable option for most individuals [60], including patients with (pre)diabetes [57, 61]. Besides, walking can be easily quantified as a daily step count and self-monitored using pedometers or activity trackers.

Goal setting is pivotal to the intervention; thus, we developed a set of recommended patient goals including: (a) increasing daily step count; (b) enhancing walking cadence; and (c) interrupting prolonged bouts of sitting.

The consensus was to advise patients to boost their daily step count by at least 3,000 above their baseline, a common goal in behavioural interventions [59, 62, 63]. This increment equates to approximately 30 min of walking, assuming a pace of 100 steps per minute—a heuristic estimate for a moderate-intensity threshold [64]. This represents more than 150 min of moderate-intensity PA each week, in line with the WHO’s guidelines for adults with chronic conditions [65]. Recognising the significance of patient autonomy, if patients find the 3,000-step increase challenging, they can propose a more feasible goal, ensuring that the goal feels personally meaningful rather than externally imposed [66]. To offer added flexibility in planning, the daily step target will be translated into a weekly goal by multiplying by seven, in line with WHO guidelines providing weekly rather than daily goals [65].

To ensure that patients achieve at least moderate-intensity levels, they will be recommended to aim for a cadence of at least 100 steps per minute [64], initially in short durations, and gradually extending these periods to make this cadence habitual. For example, patients can monitor their step count for 5 min, trying to achieve at least 500 steps, ultimately aiming for 3,000 steps in 30 min [63, 67, 68]. However, if the 100 steps per minute benchmark proves challenging, they can elevate their cadence as much as comfortably possible [65].

Lastly, given the positive effect of interrupting prolonged sitting bouts on metabolic markers in (pre)diabetes patients [8–10], they will be urged to break up sitting every 30 min for at least 3 min, during which they should either walk, preferably at moderate intensities, or perform simple exercises, such as chair squats, calf raises, or walking in place.

##### Intervention components

The mHealth intervention consists of text messages implementing various BCTs, some triggered ‘just in time’ based on Fitbit activity tracker data. To tailor the mHealth intervention to individual patients and to facilitate its adoption, patients will be initially supported with regular phone counselling. GPs initiate the mHealth intervention during routine health check-ups and provide patients with the Fitbit tracker and brief PA advice. Given that self-monitoring using a simple activity tracker has been consistently demonstrated to be effective in increasing PA levels [45, 49] and that providing PA advice by GPs is considered a standard of care [69, 70], it was deemed unethical to withhold these components from control group participants. Therefore, in the ENERGISED randomised controlled trial [32], Fitbit and brief advice will also be provided to the control group participants. Additionally, this approach enables us to isolate the net effect of the mHealth intervention beyond the activity tracker effect [42].

mHealth interventions typically use smartphone apps or text messages [71]. As (pre)diabetes is associated with older age and lower socioeconomic status [72], a notable segment of (pre)diabetes patients may be unfamiliar with app usage or might not possess a smartphone. Therefore, to ensure the broad accessibility of the intervention, we opted to convey the mHealth component through simple text messages. Text messages have been successfully used in various health interventions [73], including those promoting PA [28, 71]. A recent meta-analysis of mHealth interventions found higher effectiveness of interventions including text messaging, suggesting that it can be explained by their higher intrusiveness when compared with smartphone apps’ notifications [16].

Up until now, most messaging interventions use fixed content that is neither individualised nor adapted to fluctuations in patients’ PA. Furthermore, these messages are typically sent out at pre-defined times that do not respect the ever-changing context of individual patients [28, 71]. Leveraging the latest technological advancements, messages can be delivered just in time and adapted to the immediate context and needs of patients [74]. This precision is achieved by utilising data from sensors, such as those embedded in Fitbit trackers, which offer real-time insights into a patient’s activity patterns [75]. Just-in-time adaptive interventions (JITAIs) have recently been shown as effective in enhancing PA across diverse populations [17, 18, 76]. Examples of just-in-time messages include prompts to increase walking pace triggered when the patient is actively walking or prompts to interrupt sitting when the patient has been sedentary for over 30 min. While the full potential of such intervention can be only realised with a smartphone plus mobile data plan, we’ve ensured inclusivity by accommodating patients with only a basic cell phone with text messaging capabilities. Such patients will receive an adapted version of the mHealth intervention with no just-in-time messages, but equalised in terms of the number and types of messages delivered. This inclusive approach ensures that the intervention is suitable for a diverse range of participants, including older individuals and those from lower socioeconomic backgrounds.

##### IT solution

To power the mHealth intervention, we have adapted the HealthReact system, developed at the University of Hradec Kralove [32] and compliant with rigorous data governance standards. HealthReact serves as a comprehensive platform to collect, integrate, and evaluate sensor data, particularly from devices like the Fitbit tracker. This seamless integration facilitates the triggering of just-in-time text messages based on real-time Fitbit recorded data. Researchers can select from a broad spectrum of just-in-time triggers that can be tailored to cater to individual patients’ needs. Moreover, the system provides options to set specific parameters governing the delivery of text messages, for instance, regulating the total number of daily messages, defining the minimum interval between two consecutive messages, specifying the time window during which messages are triggered, and setting the likelihood that a triggered message is actually dispatched. This level of granularity ensures that the intervention remains adaptive and patient-centric while also ensuring that participants receive an optimal number of messages.

### Phase 2: formative research

#### Methods

Focus groups were conducted at the premises of two general practices participating in the ENERGISED trial, led by a male PA researcher with PhD and MD degrees (TV) who had no previous relationship with the participants. These focus groups comprised pre(diabetes) patients conveniently sampled from the practices by the respective GP: 7 patients (3 women, age range 53 to 66 years) from the first practice and 7 patients (1 woman, age range 63 to 78 years) from the second. The GPs welcomed the participants, then left and were not present during the focus groups that lasted 55 and 70 min, respectively. As a token of appreciation, participants were given a 20-EUR voucher.

The objective of the focus groups was to refine the key conceptual aspects developed in the previous phase, ensuring the intervention is tailored specifically to the needs and preferences of patients with (pre)diabetes. The topic guide (Additional file 2) included questions about participants’ preferred PA, patterns of sedentary behaviour, and their experiences with using activity trackers and mobile apps.

The focus groups were audio recorded and transcribed verbatim by an independent transcriber. Analysis used thematic analysis with systematic data coding to identify significant patterns and themes. A female qualitative researcher with a PhD degree (KJ) thoroughly read the transcripts, generated initial codes and grouped the codes into potential themes using NVivo software. Themes were reviewed and refined by a second researcher (TV). The analysis was both inductive, driven by the patients’ accounts, and deductive, shaped by conceptual aspects identified in phase 1.

#### Results

The formative research provided a nuanced understanding of the preferences and challenges faced by individuals with (pre)diabetes regarding PA. These insights informed the customisation of our intervention. Unfortunately, individual participants could not be identified from these focus group transcriptions, so the individual age and gender of those providing quotes cannot be given in this section.

##### Behaviour change techniques

Goal setting and regular review were supported by the focus group discussions. The participants’ acknowledgement of the motivational impact of setting and achieving PA targets aligns with our intervention’s emphasis on goal setting: “My friend uses a smartwatch to monitor his steps. He’ll notice if he’s only at 8,000 steps and say, ‘I need to reach at least 10,000 steps today,’ and then he’s up and off to achieve it.” This quote illustrates the motivational power of personal goals for behaviour change, a central element of our intervention design.

Feedback on behaviour emerged as a crucial BCT. Participants expressed a preference for feedback that was both affirming and instructive. One participant looked forward to positive reinforcement: “A text message that praises my day’s efforts in the evening and offers encouragement for the next day would be welcome.” Another participant emphasised the importance of reflective feedback to inform future actions: “I’d like an evening summary that evaluates my day, suggesting what I should start or continue doing the next day.” These insights support our intervention’s strategy of providing text messages with tailored feedback to help patients understand their progress and plan subsequent activities.

The concept of social comparison as a BCT elicited mixed reactions. Some participants saw value in a competitive edge: “They have a friendly competition over who was more active, who ran the most, who cycled the most. It certainly motivates.” This suggests that for some, comparing activities with others can be a strong motivator. Conversely, another perspective emphasised self-referenced progress: “I believe that self-comparison is key to personal progress, especially at this age.“, indicating a preference for personal benchmarks over external competition. Given these divergent views, we decided not to include social comparison in our intervention to avoid the potential negative effects of competition and to focus on individual self-improvement, which aligned with our goal of fostering intrinsic motivation.

The focus groups highlighted the importance of understanding the health consequences of PA: “I’m aware that we should all be more active and that I need to lose weight.” This acknowledgement supports the inclusion of educational text messages to inform patients about the health implications of their PA behaviours.

##### Walking as the primary mode of PA

The focus group discussions provided strong support for walking as the central PA in our intervention. Participants frequently cited walking as a preferred and accessible form of exercise. One participant’s experience highlighted that despite physical health barriers, walking was still seen as a manageable activity to increase: “I do walk and try to maintain a fast pace, but with the weight I’ve gained, even a quick 200-meter walk to the bus leaves me struggling to breathe.” Another participant maintained their walking routine despite unfavourable weather: “My dog ensures we go out for a walk every morning at seven, no matter if it’s raining, snowing, or freezing. We usually walk for half an hour, covering almost the entire block.” This comment not only illustrates the practicality of walking as an exercise that can be integrated into daily life but also shows how external motivation, such as pet ownership, can help overcome environmental barriers like bad weather. These insights collectively affirmed the choice of walking as the primary mode of PA for our intervention.

##### mHealth and wearables

The formative research phase underscored the potential of mHealth to engage patients with (pre)diabetes in managing their PA. The focus group participants expressed a general openness to using mobile technologies, with many indicating a willingness or interest in using mobile phones or wearables to support their PA goals. One participant articulated a positive stance towards technology: “That would be ideal for me; I’m quite fond of this technology.“, while another highlighted the need for simplicity: “I would be excited to use a pedometer. I’m considering purchasing one, provided it’s not too complex to use.” These insights validate our decision to employ mHealth as a key intervention component, ensuring that it is both accessible and user-friendly.

##### Just-in-time prompts

The concept of just-in-time prompts was well-received by the focus group participants: “When I’m sitting, and my watch alerts me, it prompts me to stand up, so I do.” This feedback validates our decision to incorporate just-in-time prompts into the intervention, utilising them as immediate nudges towards increased PA and reduced sedentary behaviour.

##### Phone counselling

The focus group discussions revealed a strong preference for personalised support, which reinforces the inclusion of phone counselling in our intervention. One participant expressed a desire for external motivation: “I would certainly value being more physically active, but it’s something I need to push myself to do, or else have someone else encourage and guide me.” Another participant echoed this sentiment, highlighting the importance of assistance in initiating a healthier lifestyle: “I know I should engage in it, and I would be really grateful for any help I can get to do so.” These statements underscore the value of human interaction in motivating patients to engage in PA and the essential role of counselling in supporting behaviour change.

In summary, the formative research underscored a clear preference for interventions that are not only personalised but also flexible, ensuring they can be adapted to the individual needs and circumstances of those with (pre)diabetes.

### Phase 3: pre-testing

#### Methods

In this phase, we utilised the conceptualisation refined in phase 2 to craft various types of text messages, each incorporating different BCTs. Each type had several specific examples, along with suggestions on how these messages would be triggered. A male PhD student (JN) contacted by phone the seven patients from the second focus group and invited them for face-to-face semi-structured interviews; all invited patients accepted the invitation and participated in the interviews. The interviews were conducted in the researcher’s office and lasted between 25 and 40 min. Participants were given a 20-EUR voucher.

The aim of these interviews was to gather feedback on the sample messages, which would then be used to refine and optimise the messages in alignment with the patients’ preferences and needs. To facilitate this, patients were presented with these sample messages (Table 1), prompting their immediate, think-aloud reactions. The interviews were audio recorded, transcribed verbatim, and subjected to thematic analysis using the same process and involving the same researcher (KJ) as in phase 2. However, unlike in phase 2, only deductive analysis was employed with the themes corresponding to the different types of messages.

**Table 1:** Behaviour change techniques underlying individual types of text messages and their examples

Text message type	Behaviour change techniques^a^	Example messages
Walk Faster	7.1 Prompts/cues 8.3 Habit formation	Walking fast benefits our health tremendously. Do you want to treat your body today? Try walking a little faster.
Stand Up	7.1 Prompts/cues 8.2 Behaviour substitution	We hadn’t seen any movement in a while - perfect time to get some exercise or take a brisk walk. You’ll benefit your body and feel great!
Goal Review	1.5 Review behaviour goal 1.6 Discrepancy between current behaviour and goal	You managed to meet 80% of your weekday target. Don’t slack off, you can easily catch up the remaining 20% over the weekend.
Feedback and Encouragement	2.2 Feedback on behaviour 10.4 Social reward	You did it! You have met over 100% of your weekly goal. Keep moving and next week we will celebrate again!
Action Plan Reminder	1.4 Action planning 8.3 Habit formation	A hearty walk will please every dog and benefit everyone’s health. Try to walk a little longer today with your friend.
Health Education	5.1 Information about health consequences	Regular short breaks from sitting (e.g., 2 min of walking every 30 min) have a beneficial health effect. So, get up and exercise/walk!

^a^ The Behaviour Change Techniques were coded using the taxonomy by Michie et al. [44]

#### Results

Building on the insights from formative research, we developed a series of text messages tailored to leverage specific BCTs (Table 1). The types of messages were as follows:

##### Walk faster: just-in-time prompts to increase walking pace

Participants responded positively to prompts that encouraged a faster walking pace while they were actually walking. The just-in-time nature of these messages was generally deemed crucial for their effectiveness. One participant expressed enthusiasm for the motivational aspect (Male, 63 years): “Certainly, if it provides motivation, I’d strive to reach the ‘excellent, keep going’ point. That’s the purpose of our walks, to be meaningful.”

##### Stand up: just-in-time prompts to interrupt sitting

Text messages to interrupt prolonged sitting were seen as potentially very effective. Participants valued the reminder to break their sedentary behaviour, for example, during work hours (Female, 63 years): “I can see myself doing more during work. It would fit well with my routine. But it’s challenging to remember to stand up, so I’d welcome that notification.”

##### Goal review: an interim review of the patient’s weekly step goal on friday evening

The text message with an interim review of weekly step goals was met with positive feedback, as participants valued the opportunity to reflect on their activity levels. One participant appreciated the self-monitoring aspect, recognising it as a tool for self-improvement (Male, 63 years): “It’s a useful overview. It shows what you’ve accomplished and what’s left to do, giving you a chance to catch up.” This feedback underscores the importance of such notifications in enabling patients to identify when they are falling short of their weekly targets, providing them with the motivation to increase their efforts in the remaining days of the week.

##### Feedback and encouragement: Sunday evening feedback on the patient’s weekly performance and encouragement for the upcoming week

Most participants valued the text message with feedback on their weekly performance, seeing it as a motivator for the upcoming week (Male, 69 years): “It’s beneficial to have a weekly summary… I can aim to meet or exceed it in the next week,” suggesting that reflective feedback can inspire continued or increased effort. Yet, not everyone was persuaded by the numbers, with one participant stating (Male, 65 years): “My wife might mention, ‘We’ve walked 4,000 steps,’ and I’d respond, ‘That’s irrelevant to me.’ I walk as much as I need, whether it’s 500 steps or 5,000,” indicating a preference for intuitive rather than quantified activity. Despite such views, the consensus leaned towards the usefulness of weekly performance feedback, affirming its inclusion in the intervention.

##### Action plan reminder: reminders of the action plan adapted to specific plans of each patient

Participants were instructed to suggest their own plans for how and when they wished to incorporate walking into their daily routine (e.g., walking a dog at 7 a.m. or walking home from work at 4 p.m.). The Action Plan Reminder message was then tailored to their individual plans. The desire for tailored messages was evident, with participants suggesting integration with daily routines (Female, 63 years): “If it’s aligned with a regular activity, like brushing teeth in the morning and taking a shower in the evening, then it’s possible to set messages for those times. For instance, I walk my dog every evening at seven; that would be a perfect time for a reminder.” This feedback reinforced the importance of personalising the intervention.

While most message types received positive feedback from participants, the reception of just-in-time messages suggesting an extension of walking distance was mixed. Some participants found them motivating (Female, 63 years): “Absolutely. An extra block would be manageable.” However, others expressed neutral or negative views (Male, 67 years): “I could extend my walk through the village and back. But with heavy shopping, I don’t know,” and (Male, 65 years): “Walking around the house… no, that feels odd.” Consequently, we decided to exclude this type of prompt from the intervention.

Finally, determining the optimal frequency of text messages was crucial to maintaining motivation without causing annoyance. Participants’ preferences varied widely, with some expressing indifference (Male, 65 years): “I don’t know, I don’t care,” while others specified a range (Male, 69 years): “Ideally one or two a day and no more than 10 a week,” and some were open to frequent prompts (Female, 63 years): “Even 6 to 7 a day wouldn’t bother me.” Through this feedback, it became apparent that about 10 notifications per week would be the upper limit to ensure the messages remained a welcome nudge rather than a nuisance.

### Phase 4: piloting

#### Methods

We developed a pilot version of the mHealth intervention and tested it with patients who had prediabetes or uncomplicated type 2 diabetes, were not on insulin therapy, and were regular mobile phone users, meeting the ENERGISED trial eligibility criteria (detailed in Additional file 1). Recruitment was conducted by collaborating GPs as outlined in the ENERGISED trial protocol [32]. In brief, we compiled a list of all patients with (pre)diabetes from participating general practices’ computerised medical records. A random selection of 24 patients was then made from these lists, and GPs introduced the study to all eligible patients opportunistically during routine health check-ups. This process resulted in the recruitment of 10 male patients, 4 with prediabetes and 6 with diabetes, aged between 40 and 76 years. The patients were equipped with the Fitbit Inspire 2 activity tracker [77] and instructed to maintain their typical PA for one week, using the tracker to establish their baseline steps. Subsequently, a researcher contacted them by phone, assisting them in synchronising their tracker with a Fitbit account accessible to the researchers. During this call, a daily step goal was negotiated, and opportunities for integrating walking into their daily routines were discussed, similarly as described in the Final mHealth intervention section. The information collected from this conversation was instrumental in setting up and tailoring the pilot mHealth intervention.

The pilot phase lasted two weeks, during which we monitored the number of text messages patients received. After these two weeks, the same researcher reached out to the patients for brief semi-structured interviews to gather feedback on the intervention’s usability and any potential areas for improvement. Specifically, we asked patients about the frequency, timing, and content of the messages. Patients’ responses and comments were noted during and immediately after the call and were systematically categorised by one of the researchers (TV), who also counted the number of responses per category. Patients participating in the pilot were allowed to keep the Fitbit tracker.

#### Results

During the pilot, patients received an average of 9.2 ± 10.6 text messages weekly. Five felt the frequency of messages was excessive, and two that they were sometimes sent too closely together. Three patients found the timing of the just-in-time prompts to be ill-suited; for instance, some received prompts to increase their walking cadence after completing their walk. Three participants also expressed inconvenience with receiving prompts to interrupt sitting during work hours when it wasn’t feasible. Lastly, five patients felt that the message content was repetitive.

To address these issues, we implemented several intervention refinements. Specifically, we adjusted the probability of dispatching certain text messages and reduced the maximum number of just-in-time daily prompts (Table 2). This ensures that most patients will receive between three to six messages weekly, with only occasional weeks exceeding ten messages. Additionally, we fine-tuned the parameters for triggering just-in-time prompts related to walking cadence, minimising the likelihood of sending a prompt once walking had finished. However, this adjustment potentially leads to infrequent prompts for patients who engage in minimal walking. To address concerns about receiving prompts to interrupt sitting during work hours, we restricted these prompts to a window between 4 pm and 8 pm. Lastly, to diversify the content and reduce repetitiveness, we crafted multiple text variations for each message type.

**Table 2:** Overview of text messages comprised in the intervention and their triggering rules

Text message type	Day	Maximum number per day	Time window	Trigger	Probability^a^ of being dispatched
Walk Faster	Daily	2 (> 60 min in between)	8 am– 8 pm	5 min of 60–100 steps/min	50%
*Walk Faster - Adapted* ^b^	Daily	1	8 am– 8 pm	Randomly within the time window	15%
Stand Up	Daily	1	4 pm– 8 pm	30 min of 0 steps/min and recorded heart rate	50%
*Stand Up - Adapted* ^c^	Daily	1	4 pm– 8 pm	Randomly within the time window	15%
Goal Review	Friday	1	8 pm– 10 pm	Randomly within the time window	50%
Feedback and Encouragement	Sunday	1	6 pm– 8 pm	Randomly within the time window	50%
Action Plan Reminder	Individual	1	Individual	Randomly within the time window	50%
Health Education	Tuesday	1	6 pm– 8 pm	Randomly within the time window	50%

^a^ Probability that a triggered message is actually dispatched, i.e., sent to the patient. ^b^ The adapted Walk Faster messages are only sent to patients in groups B and C who do not receive just-in-time Walk Faster messages due to irregularity of their Fitbit syncing pattern. ^c^ The adapted Stand Up messages are only sent to patients in group C who do not receive just-in-time Stand Up messages due to none or very limited syncing of their Fitbit tracker

### Final mHealth intervention

The final intervention comprises six types of text messages, each embodying different BCTs. The individual types, examples of text messages, BCTs utilised, triggering rules, and probability of their dispatch are detailed in Tables 1 and 2. The detailed implementation of the intervention within primary care settings is thoroughly described in the previously published ENERGISED trial protocol [32].

Walk Faster and Stand Up messages are triggered just in time by specific physical behaviour events as detected by Fitbit sensors: 5 min with a step count ranging from 60 to 100 (allowing for one outlier minute below and two above the range) between 8 am and 8 pm for Walk Faster, and 30 min with zero steps while detecting heart rate (to confirm wear) between 4 and 8 pm for Stand Up messages. To prevent overwhelming patients, the frequency of these messages is capped at one per day for Stand Up and two per day for Walk Faster (with a minimum interval of 60 min between them).

Action Plan Reminder messages are triggered according to individual participants’ routines once or more times per week. Goal Review, Feedback and Encouragement, and Health Education messages are triggered at predetermined times once a week, separated throughout the week. Goal Review is triggered on Friday evenings between 8 and 10 pm, allowing participants time over the weekend to catch up. Feedback and Encouragement messages are triggered on Sunday evenings between 6 and 8 pm, offering a review of the past week and motivation for the week ahead. Health Education messages are triggered on Tuesdays between 6 and 8 pm.

Each message’s dispatch is determined by a randomisation algorithm, which decides with a given probability (Table 2) whether the message is actually dispatched to the patient. For instance, the 50% probability of the Goal Review means that the message is triggered every Friday but only dispatched every other week on average. This randomisation not only further limits the weekly text message count but also facilitates the future evaluation of each message’s immediate impact on objectively measured PA levels using a micro-randomised design.

For each message type, there are various text versions from which one is randomly selected (Table 1). Furthermore, the content of Action Plan Reminder messages is tailored to each participant’s individual plans. Goal Review and Feedback and Encouragement messages are also personalised, reflecting each participant’s step count from recent days.

Of note, all standard notifications and prompts typically delivered by the Fitbit wearable and its accompanying app are deliberately deactivated for both intervention and control participants to ensure that they do not interfere with our intervention.

#### Adapted intervention

For optimal functioning of the intervention, patients require a smartphone compatible with the HealthReact and Fitbit apps (Android 9.0 or iOS 15.0 and later as of November 2023), along with a mobile data plan for continuous internet connectivity. These patients comprise Group A. For participants lacking such resources, the intervention is modified based on the reliability of their Fitbit data syncing:

Group B: Participants in this group who don’t sync their data continuously throughout the day but do sync regularly every day, usually in the afternoon and evening hours (often those without a mobile data plan but with a reliable Wi-Fi connection at home), receive additional time-based Walk Faster messages. This approach compensates for their lack of just-in-time Walk Faster messages due to their syncing patterns.

Group C: Participants who either sync irregularly or not at all (including those with basic cell phones instead of smartphones) are provided additional time-based Walk Faster and Stand Up messages to make up for the absence of just-in-time Walk Faster and Stand Up messages. Furthermore, in these cases, Goal Review and Feedback and Encouragement messages cannot be personalised due to missing recent step count data. Therefore, they receive non-personalised messages that remind them to review their goals and provide encouragement.

Importantly, the adapted intervention is equalised in terms of the number and types of messages delivered. This equalisation is achieved by triggering the adapted time-based Walk Faster and Stand Up messages for Groups B and C only once per day, with the probability of these messages being dispatched set at 15%.

#### Procedures and counselling

During the baseline visit, all patients (intervention and control) receive a Fitbit Inspire 2 activity tracker from their GP, along with brief PA advice complemented by an educational leaflet and a prescription for PA. Additionally, patients are instructed to maintain their usual PA levels for one more week while wearing the Fitbit to establish their baseline steps.

Approximately one to two weeks later, intervention patients are contacted by phone by a counsellor who assists them in setting individual goals and devising an action plan (e.g., walking a dog for 30 min on three specific days of the week). The counsellor then inputs this information into the HealthReact system to tailor the Action Plan Reminder messages and enable personalisation of the Goal Review and Feedback and Encouragement messages.

In subsequent calls at months 1 to 6 (lead-in phase) to intervention patients, the counsellor supports patients in reviewing their step goals and action plans, employing various BCTs to facilitate goal achievement. During these calls, the counsellor can adjust the mHealth intervention to adapt to the changing needs of the patients. For instance, if patients consistently achieve their step goal, the counsellor may challenge them to increase it. The counsellor also assists patients with technical issues related to the intervention.

From month 7 onwards (maintenance phase), patients no longer receive phone counselling but continue to receive text messages for an additional six months, until month 12, as previously described.

#### Intervention monitoring

**Fig. 1:**
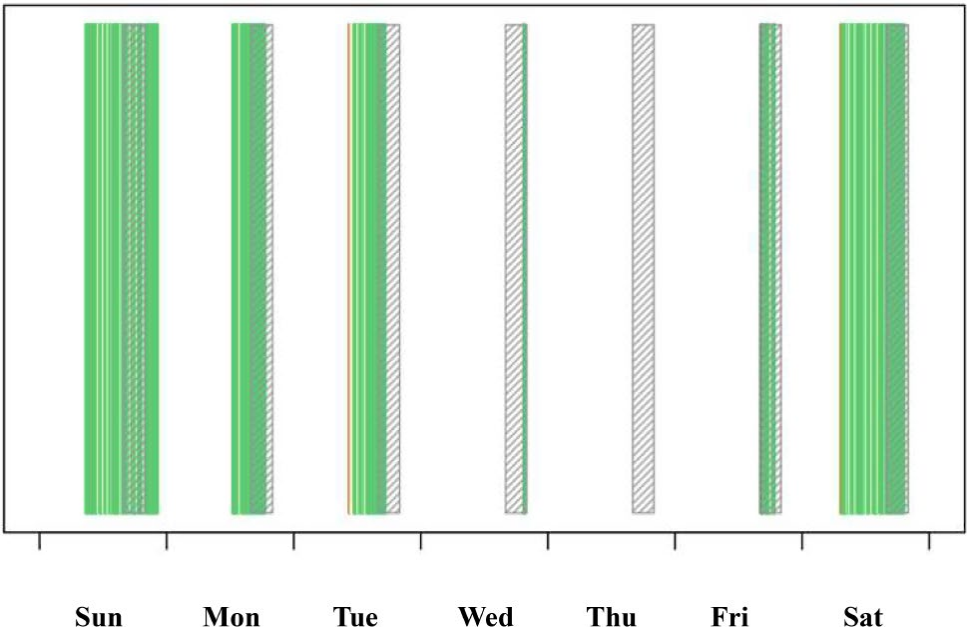
A sample of the weekly report of a patient’s Fitbit syncing pattern. The vertical green lines represent individual Fitbit syncs. The compact green area signifies regular syncing (approx. every 15 min). The hatched area marks time periods from 4 to 8 pm when just-in-time Stand Up text messages are triggered. This specific patient would be classified as Group B: irregular syncing, mostly in the afternoon and during weekends, probably only when connected to the Wi-Fi at home. Despite the irregular sync, the patient would likely receive several just-in-time Stand Up messages per week (assuming she spent 30 min sitting), but hardly any just-in-time Walk Faster messages. Hence, her classification as Group B, which receives adapted Walk Faster messages independent of Fitbit data

The phone counsellors will review regular weekly reports of their patients’ Fitbit syncing patterns (Fig. 1). Should a patient’s syncing reliability decline, they initially receive a text message from the phone counsellor, prompting regular syncing. If this reminder proves ineffective, the counsellor addresses the issue in the subsequent scheduled call. Persistent syncing challenges may necessitate reassigning the patient to a group with a lower syncing requirement (e.g., from Group A to Group B, or Group B to Group C). Conversely, if patients in Group C or Group B demonstrate improved syncing consistency, surpassing their current group’s requirements, they are upgraded to a more appropriate group (Group B or Group A, respectively). This dynamic approach ensures each participant benefits from the most effective version of the intervention, tailored to their specific mobile phone capabilities and internet access.

## Discussion

When developing mHealth interventions, a participatory approach involving patients is critical to enhance the intervention’s relevance and ensure its adaptability to real-world settings [78]. The participatory approach also demonstrated its value in the development of our mHealth intervention. Initially, key components such as walking as the primary mode of physical activity, the provision of activity trackers, and the implementation of just-in-time prompts were conceived during the first conceptualisation phase, which relied on published evidence and expert opinions. However, it was the subsequent involvement of patients in the development process that truly affirmed and refined these components. For instance, feedback from participants underscored the importance of walking for its accessibility and potential for seamless integration into daily routines. Another example is the inclusion of phone counselling support, which was particularly valued for its personal touch and ability to facilitate the initial adoption of the intervention.

Moreover, the participatory phases enabled us to refine the intervention based on patient suggestions, leading to significant enhancements. The frequency of text messages, the customisation of Action Plan Reminder messages, and the individualisation of Feedback and Encouragement and Goal Review messages were all adjusted to better meet the needs and preferences of the target population. Additionally, to maintain engagement and avoid monotony, we introduced variations in the message content based on patient feedback.

Conversely, the participatory approach also led to the exclusion of certain features initially considered for inclusion. Based on patient feedback, we decided against incorporating social comparison elements and suggestions for extending walking distances, as they were not favoured by the participants. This iterative process of inclusion and exclusion highlights the strength of involving patients directly in the development of health interventions.

Furthermore, some patient suggestions introduced specific limitations, such as the decision to deliver Stand Up messages only from 4 pm to 8 pm to minimise interference with work routines. While this decision was made to enhance the practicality of the intervention, it also illuminated the nuanced balance between customisation and efficacy.

These examples illustrate how the participatory approach not only validated the initial conceptualisation of the intervention but also led to its substantial refinement. This process ensured that the final intervention was not only grounded in evidence but also resonant with the needs and preferences of the target population.

### Study strengths

Applying the ‘mHealth development and evaluation framework’, including active participation of the target audience, to the development of our intervention endowed it with several strengths, essential for its potential success.

First, we identified walking as the primary mode of PA due to its accessibility and potential for seamless integration into daily routines [57]. Recognising that merely accumulating steps might be insufficient for significant health benefits [59], we emphasised walking cadence to reach a threshold indicative of moderate PA. Additionally, our intervention focuses on interrupting prolonged periods of sitting, a behaviour particularly detrimental to patients with (pre)diabetes [8–10].

Second, central to our intervention are mHealth technologies and wearable activity trackers, which offer sustainable solutions scalable to a broad population of (pre)diabetes patients within primary care [79]. Just-in-time prompts designed to increase walking cadence and interrupt prolonged sitting can be particularly effective, as they deliver timely, context-specific nudges [17, 18]. To broaden accessibility, including for those with limited technology literacy, the mHealth intervention is delivered in the form of text messages [16, 71]. Furthermore, we developed an adapted version of the intervention for patients without a mobile data plan or those with only basic cell phones, ensuring inclusivity.

Third, the involvement of patients in the intervention’s development highlighted the importance of tailoring and personalisation. Consequently, most text messages were designed to be individualised for each patient. For instance, Action Plan Reminder messages can be customised according to each participant’s specific routines and preferences. Furthermore, Feedback and Encouragement and Goal Review messages leverage individual goals and real-time performance data from Fitbit to provide a personalised experience for each participant. To maintain engagement and avoid monotony, we produced several variations of the message content and carefully regulated the number of messages per week to prevent intervention fatigue.

Finally, phone counselling during the lead-in phase of the intervention plays a pivotal role [42]. This personal touch not only facilitates the initial adoption of the intervention but also provides necessary support and guidance, ensuring participants are comfortable and engaged with the technology and the overall program. This combination of technological innovation and human interaction was instrumental in creating an effective, patient-centric intervention to enhance PA in (pre)diabetes patients.

### Study limitations

Our study has limited generalizability due to reliance on a small sample of patients with (pre)diabetes who participated in the development of the mHealth intervention. Additionally, the gender imbalance in our participant group, with a predominance of male participants, further constrains generalizability. This selective group may not fully represent the broader population of all patients with (pre)diabetes, especially those less inclined to use technology-based solutions. Furthermore, while efforts were made to enhance accessibility, the intervention’s reliance on text messaging and wearable technology presupposes a certain level of technological literacy that may not be universally present.

Furthermore, our development process, guided by the ‘mHealth development and evaluation framework’, did not involve patient partners in the initial conceptualisation phase, which relied on published evidence and expert opinions [38–40]. This approach was chosen to establish a strong evidence-based foundation for the intervention. However, we acknowledge this as a limitation, recognising the value of patient involvement from the earliest stages of intervention development.

Additionally, while our multidisciplinary research team engaged in comprehensive discussions to reach a consensus on the key conceptual aspects of the intervention, we did not employ any structured approach, such as a Delphi method, in phase 1. The absence of this formal consensus method may have limited the systematic integration of diverse expert opinions and could be considered a limitation of our methodology. In addition, the absence of a structured approach precluded detailed reporting of individual team members’ specific feedback in phase 1, thereby diminishing the transparency of findings derived from this consultative process.

Another limitation relates to the use of Fitbit wearables. While they are affordable and user-friendly, Fitbit devices only sync with their server approximately every quarter of an hour. Consequently, the data triggering the just-in-time prompts can be delayed by up to 15 minutes (assuming a constant internet connection), leading to prompts that are ‘not-quite-in-time,’ as detailed in the ‘Phase 4: piloting’ section. To mitigate this, our intervention only considers data immediately preceding the sync. However, this workaround potentially results in missed triggers, especially for patients who engage in minimal walking, leading to less frequent delivery of Walk Faster messages.

Lastly, based on patient feedback, we opted to deliver Stand Up messages only from 4 pm to 8 pm to minimise negative interference with participants’ work routines and prevent annoyance. However, restricting prompts to interrupt sitting to this specific time frame may limit the efficacy of the intervention, as it doesn’t address prolonged sitting during a significant portion of the day.

## Conclusions

The development of our mHealth intervention, rooted in a participatory design approach, underscores the importance of involving patients in creating behavioural interventions tailored to their specific needs. The incorporation of just-in-time prompts, which leverage real-time data from wearable devices, represents a significant advancement in delivering personalised and context-sensitive PA interventions for patients with (pre)diabetes. Should this approach prove effective in the ongoing ENERGISED randomised controlled trial within a primary care setting, it could significantly aid GPs in guiding patients towards increased PA and reduced sedentary lifestyles. The integration of mHealth tools offers a promising solution for GPs to overcome time constraints and enhance their capacity for behavioural counselling, leveraging the trusted patient-GP relationship. In doing so, GPs can provide continuous, personalised guidance, crucial for the management of (pre)diabetes and potentially adaptable for other health conditions in routine primary care settings.

### Electronic supplementary material

Below is the link to the electronic supplementary material.

Supplementary Material 1

Supplementary Material 2

## Data Availability

The datasets generated during the current study are available from the corresponding author upon reasonable request.

## References

[1] Sun H, Saeedi P, Karuranga S, Pinkepank M, Ogurtsova K, Duncan BB (2022). IDF Diabetes Atlas: Global, regional and country-level diabetes prevalence estimates for 2021 and projections for 2045. Diabetes Res Clin Pr.

[2] Rooney MR, Fang M, Ogurtsova K, Ozkan B, Echouffo-Tcheugui JB, Boyko EJ (2023). Global prevalence of Prediabetes. Diabetes Care.

[3] Cosentino F, Grant PJ, Aboyans V, Bailey CJ, Ceriello A, Delgado V (2019). 2019 ESC guidelines on diabetes, pre-diabetes, and cardiovascular diseases developed in collaboration with the EASD. Eur Heart J.

[4] Kanaley JA, Colberg SR, Corcoran MH, Malin SK, Rodriguez NR, Crespo CJ (2022). Exercise/Physical activity in individuals with type 2 diabetes: a Consensus Statement from the American College of Sports Medicine. Med Sci Sports Exerc.

[5] Geidl W, Schlesinger S, Mino E, Miranda L, Pfeifer K (2020). Dose–response relationship between physical activity and mortality in adults with noncommunicable diseases: a systematic review and meta-analysis of prospective observational studies. Int J Behav Nutr Phy.

[6] Jadhav RA, Hazari A, Monterio A, Kumar S, Maiya AG (2017). Effect of physical activity intervention in Prediabetes: a systematic review with Meta-analysis. J Phys Act Health.

[7] Yates T, Haffner SM, Schulte PJ, Thomas L, Huffman KM, Bales CW (2014). Association between change in daily ambulatory activity and cardiovascular events in people with impaired glucose tolerance (NAVIGATOR trial): a cohort analysis. Lancet.

[8] Yates T, Edwardson CL, Celis-Morales C, Biddle SJH, Bodicoat D, Davies MJ (2018). Metabolic effects of breaking prolonged sitting with standing or light walking in older South asians and White europeans: a randomized Acute Study. J Gerontol Biol Sci.

[9] Loh R, Stamatakis E, Folkerts D, Allgrove JE, Moir HJ (2019). Effects of interrupting prolonged sitting with physical activity breaks on blood glucose, insulin and triacylglycerol measures: a systematic review and Meta-analysis. Sports Med.

[10] Dempsey PC, Larsen RN, Sethi P, Sacre JW, Straznicky NE, Cohen ND (2016). Benefits for type 2 diabetes of interrupting prolonged sitting with brief bouts of light walking or simple resistance activities. Diabetes Care.

[11] Zhao G, Ford ES, Li C, Mokdad AH (2008). Compliance with physical activity recommendations in US adults with diabetes. Diabet Med.

[12] Barker J, Byrne KS, Doherty A, Foster C, Rahimi K, Ramakrishnan R (2019). Physical activity of UK adults with chronic disease: cross-sectional analysis of accelerometer-measured physical activity in 96 706 UK Biobank participants. Int J Epidemiol.

[13] Mortensen SR, Skou ST, Brønd JC, Ried-Larsen M, Petersen TL, Jørgensen LB (2023). Detailed descriptions of physical activity patterns among individuals with diabetes and prediabetes: the Lolland-Falster Health Study. BMJ Open Diabetes Res Care.

[14] Hendrick P, Zihao H, Mönninghoff A, Kramer JN, Hess AJ, Ismailova K (2021). Long-term effectiveness of mHealth Physical Activity interventions: systematic review and Meta-analysis of Randomized controlled trials. J Med Internet Res.

[15] Müller AM, Maher CA, Vandelanotte C, Hingle M, Middelweerd A, Lopez ML (2018). Physical activity, sedentary behavior, and Diet-related eHealth and mHealth Research: bibliometric analysis. J Med Internet Res.

[16] Laranjo L, Ding D, Heleno B, Kocaballi B, Quiroz JC, Tong HL (2021). Do smartphone applications and activity trackers increase physical activity in adults? Systematic review, meta-analysis and metaregression. Brit J Sport Med.

[17] Nahum-Shani I, Smith SN, Spring BJ, Collins LM, Witkiewitz K, Tewari A (2016). Just-in-Time adaptive interventions (JITAIs) in Mobile Health: Key Components and Design principles for Ongoing Health Behavior support. Ann Behav Med.

[18] Hardeman W, Houghton J, Lane K, Jones A, Naughton F (2019). A systematic review of just-in-time adaptive interventions (JITAIs) to promote physical activity. Int J Behav Nutr Phys Act.

[19] Matthew-Maich N, Harris L, Ploeg J, Markle-Reid M, Valaitis R, Ibrahim S (2016). Designing, Implementing, and Evaluating Mobile Health Technologies for managing chronic conditions in older adults: a scoping review. Jmir Mhealth Uhealth.

[20] Aguilera A, Figueroa CA, Hernandez-Ramos R, Sarkar U, Cemballi A, Gomez-Pathak L (2020). mHealth app using machine learning to increase physical activity in diabetes and depression: clinical trial protocol for the DIAMANTE Study. Bmj Open.

[21] Schroé H, Crombez G, Bourdeaudhuij ID, Dyck DV (2022). Investigating when, which, and why users stop using a Digital Health Intervention to promote an active lifestyle: secondary analysis with a focus on health action process Approach–Based Psychological determinants. Jmir Mhealth Uhealth.

[22] Mayberry LS, Lyles CR, Oldenburg B, Osborn CY, Parks M, Peek ME (2019). mHealth interventions for disadvantaged and vulnerable people with type 2 diabetes. Curr Diabetes Rep.

[23] Orrow G, Kinmonth A-L, Sanderson S, Sutton S (2012). Effectiveness of physical activity promotion based in primary care: systematic review and meta-analysis of randomised controlled trials. BMJ.

[24] Kettle VE, Madigan CD, Coombe A, Graham H, Thomas JJC, Chalkley AE (2022). Effectiveness of physical activity interventions delivered or prompted by health professionals in primary care settings: systematic review and meta-analysis of randomised controlled trials. BMJ.

[25] Hébert ET, Caughy MO, Shuval K (2012). Primary care providers’ perceptions of physical activity counselling in a clinical setting: a systematic review. Brit J Sport Med.

[26] Wändell PE, de Waard A-KM, Holzmann MJ, Gornitzki C, Lionis C, de Wit N (2018). Barriers and facilitators among health professionals in primary care to prevention of cardiometabolic diseases: a systematic review. Fam Pract.

[27] Vetrovsky T, Cupka J, Dudek M, Kuthanova B, Vetrovska K, Capek V (2018). A pedometer-based walking intervention with and without email counseling in general practice: a pilot randomized controlled trial. BMC Public Health.

[28] Khunti K, Griffin S, Brennan A, Dallosso H, Davies MJ, Eborall HC (2021). Promoting physical activity in a multi-ethnic population at high risk of diabetes: the 48-month PROPELS randomised controlled trial. Bmc Med.

[29] Bondaronek P, Dicken SJ, Jennings SS, Mallion V, Stefanidou C (2022). Barriers to and facilitators of the Use of Digital Tools in Primary Care to deliver physical activity advice: Semistructured Interviews and thematic analysis. Jmir Hum Factors.

[30] Poppe L, Plaete J, Huys N, Verloigne M, Deveugele M, Bourdeaudhuij ID (2018). Process evaluation of an eHealth intervention implemented into General Practice: General practitioners’ and patients’ views. Int J Environ Res Public Health.

[31] Patel A, Schofield GM, Kolt GS, Keogh JWL (2013). Perceived barriers, benefits, and motives for physical activity: two primary-care physical activity prescription programs. J Aging Phys Act.

[32] Vetrovsky T, Kral N, Pfeiferova M, Kuhnova J, Novak J, Wahlich C (2023). mHealth intervention delivered in general practice to increase physical activity and reduce sedentary behaviour of patients with prediabetes and type 2 diabetes (ENERGISED): rationale and study protocol for a pragmatic randomised controlled trial. BMC Public Health.

[33] Bickmann P, Froböse I, Grieben C (2024). An mHealth application in German Health Care System: importance of user participation in the development process. J Med Syst.

[34] Chudyk AM, Ragheb S, Kent D, Duhamel TA, Hyra C, Dave MG (2021). Patient Engagement in the design of a Mobile Health App that supports enhanced recovery protocols for cardiac surgery: Development Study. Jmir Perioper Med.

[35] Richards T, Schroter S, Price A, Godlee F (2018). Better together: patient partnership in medical journals. BMJ.

[36] Wright CJ, Dietze PM, Crockett B, Lim MS (2016). Participatory development of MIDY (Mobile intervention for drinking in young people). BMC Public Health.

[37] Hietbrink E, Middelweerd A, van Empelen P, Preuhs K, Konijnendijk A, Oude Nijeweme-d’Hollosy W (2023). A Digital Lifestyle Coach (E-Supporter 1.0) to support people with type 2 diabetes: Participatory Development Study. Jmir Hum Factors.

[38] Fjeldsoe BS, Miller YD, O’Brien JL, Marshall AL (2012). Iterative development of MobileMums: a physical activity intervention for women with young children. Int J Behav Nutr Phys Act.

[39] Whittaker R, Whittaker R, Merry S, Merry S, Dorey E, Dorey E (2012). A development and evaluation process for mHealth interventions: examples from New Zealand. J Health Commun.

[40] Morton K, Sutton S, Hardeman W, Troughton J, Yates T, Griffin S (2015). A text-messaging and pedometer program to promote physical activity in people at high risk of type 2 diabetes: the development of the PROPELS Follow-On support program. Jmir Mhealth Uhealth.

[41] Avery L, Charman SJ, Taylor L, Flynn D, Mosely K, Speight J (2015). Systematic development of a theory-informed multifaceted behavioural intervention to increase physical activity of adults with type 2 diabetes in routine primary care: Movement as Medicine for type 2 diabetes. Implement Sci.

[42] Vetrovsky T, Borowiec A, Juřík R, Wahlich C, Śmigielski W, Steffl M (2022). Do physical activity interventions combining self-monitoring with other components provide an additional benefit compared with self-monitoring alone? A systematic review and meta-analysis. Brit J Sport Med.

[43] Compernolle S, DeSmet A, Poppe L, Crombez G, Bourdeaudhuij ID, Cardon G (2019). Effectiveness of interventions using self-monitoring to reduce sedentary behavior in adults: a systematic review and meta-analysis. Int J Behav Nutr Phy.

[44] Dyck DV, Greef KD, Deforche B, Ruige J, Bouckaert J, Tudor-Locke CE (2013). The relationship between changes in steps/day and health outcomes after a pedometer-based physical activity intervention with telephone support in type 2 diabetes patients. Heal Educ Res.

[45] Chaudhry UAR, Wahlich C, Fortescue R, Cook DG, Knightly R, Harris T (2020). The effects of step-count monitoring interventions on physical activity: systematic review and meta-analysis of community-based randomised controlled trials in adults. Int J Behav Nutr Phys Act.

[46] Bandura A (1991). Social cognitive theory of self-regulation. Organ Behav Hum Decis Process.

[47] Mair JL, Salamanca-Sanabria A, Frese B, Jakob R, Kowatsch T, Haug S (2023). Effective behavior change techniques in digital health interventions targeting non-communicable diseases: an umbrella review. Ann Behav Med.

[48] Michie S, Richardson M, Johnston M, Abraham C, Francis J, Hardeman W (2013). The behavior change technique taxonomy (v1) of 93 hierarchically clustered techniques: building an international consensus for the reporting of behavior change interventions. Ann Behav Med.

[49] Oliveira JS, Sherrington C, Zheng ERY, Franco MR, Tiedemann A (2019). Effect of interventions using physical activity trackers on physical activity in people aged 60 years and over: a systematic review and meta-analysis. Brit J Sport Med.

[50] Latham GP, Locke EA (1991). Self-regulation through goal setting. Organ Behav Hum Decis Process.

[51] McEwan D, Beauchamp MR, Kouvousis C, Ray CM, Wyrough A, Rhodes RE (2018). Examining the active ingredients of physical activity interventions underpinned by theory versus no stated theory: a meta-analysis. Health Psychol Rev.

[52] Cradock KA, ÓLaighin G, Finucane FM, Gainforth HL, Quinlan LR, Ginis KAM (2017). Behaviour change techniques targeting both diet and physical activity in type 2 diabetes: a systematic review and meta-analysis. Int J Behav Nutr Phys Act.

[53] Direito A, Carraça E, Rawstorn J, Whittaker R, Maddison R (2016). mHealth technologies to influence physical activity and sedentary behaviors: Behavior Change techniques, systematic review and Meta-analysis of Randomized controlled trials. Ann Behav Med.

[54] Carraça E, Encantado J, Battista F, Beaulieu K, Blundell J, Busetto L (2021). Effective behavior change techniques to promote physical activity in adults with overweight or obesity: a systematic review and meta-analysis. Obes Rev.

[55] Duff OM, Walsh DM, Furlong BA, O’Connor NE, Moran KA, Woods CB (2017). Behavior change techniques in physical activity eHealth interventions for people with Cardiovascular Disease: systematic review. J Med Internet Res.

[56] Simões P, Silva AG, Amaral J, Queirós A, Rocha NP, Rodrigues M, Features (2018). Behavioral change techniques, and quality of the most popular mobile apps to measure physical activity: systematic search in App stores. Jmir Mhealth Uhealth.

[57] Moghetti P, Balducci S, Guidetti L, Mazzuca P, Rossi E, Schena F (2020). Walking for subjects with type 2 diabetes: a systematic review and joint AMD/SID/SISMES evidence-based practical guideline. Nutr Metab Cardiovasc Dis.

[58] Oja P, Kelly P, Murtagh EM, Murphy MH, Foster C, Titze S (2018). Effects of frequency, intensity, duration and volume of walking interventions on CVD risk factors: a systematic review and meta-regression analysis of randomised controlled trials among inactive healthy adults. Br J Sports Med.

[59] Vetrovsky T, Siranec M, Frybova T, Gant I, Svobodova I, Linhart A (2024). Lifestyle walking intervention for patients with heart failure with reduced ejection fraction: the WATCHFUL trial. Circulation.

[60] Morris JN, Hardman AE (1997). Walking to Health. Sports Med.

[61] Funk M, Taylor EL (2013). Pedometer-based walking interventions for free-living adults with type 2 diabetes: a systematic review. Curr Diabetes Rev.

[62] Tudor-Locke C, Craig CL, Aoyagi Y, Bell RC, Croteau KA, Bourdeaudhuij ID (2011). How many steps/day are enough? For older adults and special populations. Int J Behav Nutr Phys Act.

[63] Harris T, Kerry SM, Limb ES, Victor CR, Iliffe S, Ussher M (2017). Effect of a primary care walking intervention with and without nurse support on physical activity levels in 45- to 75-Year-Olds: the Pedometer and Consultation evaluation (PACE-UP) cluster randomised clinical trial. Plos Med.

[64] Tudor-Locke C, Ducharme SW, Aguiar EJ, Schuna JM, Barreira TV, Moore CC (2020). Walking cadence (steps/min) and intensity in 41 to 60-year-old adults: the CADENCE-adults study. Int J Behav Nutr Phys Act.

[65] Bull FC, Al-Ansari SS, Biddle S, Borodulin K, Buman MP, Cardon G (2020). World Health Organization 2020 guidelines on physical activity and sedentary behaviour. Brit J Sport Med.

[66] Huisman S, Maes S, Gucht VJD, Chatrou M, Haak HR (2010). Low goal ownership predicts Drop-out from a weight intervention study in overweight patients with type 2 diabetes. Int J Behav Med.

[67] Marshall SJ, Levy SS, Tudor-Locke CE, Kolkhorst FW, Wooten KM, Ji M (2009). Translating physical activity recommendations into a pedometer-based step goal 3000 steps in 30 minutes. Am J Prev Med.

[68] Yates T, Davies M, Gorely T, Bull F, Khunti K (2009). Effectiveness of a pragmatic education program designed to promote walking activity in individuals with impaired glucose tolerance: a randomized controlled trial. Diabetes Care.

[69] Karen I, Svacina S (2020). Diabetes mellitus.

[70] Karen I, Svacina S, Prediabetes. Prague: Czech Society of General Practice; 2016.

[71] Smith DM, Duque L, Huffman JC, Healy BC, Celano CM (2019). Text message interventions for physical activity: a systematic review and Meta-analysis. Am J Prev Med.

[72] Kyrou I, Tsigos C, Mavrogianni C, Cardon G, Stappen VV, Latomme J et al. Sociodemographic and lifestyle-related risk factors for identifying vulnerable groups for type 2 diabetes: a narrative review with emphasis on data from Europe. BMC Endocr Disord. 2020;20 Suppl 1:134. 10.1186/s12902-019-0463-3 PMC7066728 32164656

[73] Head KJ, Noar SM, Iannarino NT, Harrington NG (2013). Efficacy of text messaging-based interventions for health promotion: a meta-analysis. Soc Sci Med.

[74] Park J, Kim M, Mistiri ME, Kha R, Banerjee S, Gotzian L (2023). Advancing understanding of just-in-Time States for supporting physical activity (Project JustWalk JITAI): protocol for a System ID Study of just-in-Time adaptive interventions. Jmir Res Protoc.

[75] Mair JL, Hayes LD, Campbell AK, Buchan DS, Easton C, Sculthorpe N (2022). A personalized smartphone-delivered just-in-time adaptive intervention (JitaBug) to increase physical activity in older adults: mixed methods feasibility study. Jmir Form Res.

[76] Fiedler J, Seiferth C, Eckert T, Woll A, Wunsch K (2023). A just-in-Time adaptive intervention to enhance physical activity in the SMARTFAMILY2.0 trial. Sport Exerc Perform Psychol.

[77] Delobelle J, Lebuf E, Dyck DV, Compernolle S, Janek M, Backere FD (2022). Fitbit’s accuracy to measure short bouts of physical activity and sedentary behavior: validation, sensitivity and specificity study. Preprint.

[78] Bird M, Ouellette C, Whitmore C, Li L, Nair K, McGillion MH (2020). Preparing for patient partnership: a scoping review of patient partner engagement and evaluation in research. Heal Expect.

[79] McMillan KA, Kirk A, Hewitt A, MacRury S (2016). A systematic and Integrated Review of Mobile-Based Technology to promote active lifestyles in people with type 2 diabetes. J Diabetes Sci Technol.

