# Supplementary material for: Participatory development of an mHealth intervention delivered in general practice to increase physical activity and reduce sedentary behaviour of patients with prediabetes and type 2 diabetes (ENERGISED)
Source: BMC Public Health. 2024 Mar 31;24:927. doi: 10.1186/s12889-024-18384-2 (PMC10983629; doi:10.1186/s12889-024-18384-2)
Supplement: Supplementary file 1 — Supplementary Material 1 [file 12889_2024_18384_MOESM1_ESM.docx]

Additional file 1

# Patient eligibility criteria for the ENERGISED trial

To be eligible for the trial, patients must meet the following inclusion criteria at randomisation: (1) Diagnosis of prediabetes or type 2 diabetes according to the Czech guidelines for GPs, i.e. fasting plasma glucose 5.6–6.9 mmol/l, or 2-h plasma glucose of 7.8– 11.0 mmol/l after ingestion of 75 g of oral glucose load for the diagnosis of prediabetes, and fasting plasma glucose ≥7.0 mmol/l, or 2-h plasma glucose ≥11.1 mmol/l after ingestion of 75 g of the oral glucose load for the diagnosis of type 2 diabetes. (2) Age 18 years or older. (3) Followed for prediabetes/diabetes by a participating GP. Of note, in the Czech Republic, only uncomplicated type 2 diabetes patients with glycated haemoglobin HbA1c ≤53 mmol/mol and not taking insulin are commonly followed by a GP; other type 2 diabetes patients are usually followed by a specialist diabetologist. (4) Regular mobile phone users (not necessarily a smartphone), able and willing to answer calls and read text messages as part of the study. (5) Able and willing to wear and use a wrist-worn Fitbit activity tracker for the study duration. (6) Written informed consent provided before any assessment related to the study.

Patients are excluded from the trial if they are: (1) Unable to walk for any reason. (2) Pregnant. (3) Having a household member already recruited for this study to avoid contamination. (4) Living in a residential or nursing care home where the imposed regime could interfere with the intervention. (5) Having any co-morbid conditions that would seriously affect their adherence to the trial procedures (e.g., active malignancy; recent (<3 months) myocardial infarction, coronary artery bypass graft or cerebrovascular accident; renal disease requiring dialysis; neurological condition (e.g., Parkinson disease); cognitive impairment, or significant hearing or visual impairment; hip or knee joint replacement within three months; major surgery planned within the next 12 months).
