# Supplementary material for: Participatory development of an mHealth intervention delivered in general practice to increase physical activity and reduce sedentary behaviour of patients with prediabetes and type 2 diabetes (ENERGISED)
Source: BMC Public Health. 2024 Mar 31;24:927. doi: 10.1186/s12889-024-18384-2 (PMC10983629; doi:10.1186/s12889-024-18384-2)
Supplement: Supplementary file 2 — Supplementary Material 2 [file 12889_2024_18384_MOESM2_ESM.docx]

Additional file 1

# Topic guide for focus groups within Phase 2 (Formative research)

1. What physical activity do you usually do during the week? How much of it is at least moderate intensity?

2. When are you most likely to be sitting down? How long do you usually sit without interruption?

3. Do you have any experience with physical activity self-monitoring using an activity tracker such as a Fitbit, a smartwatch, or a mobile app?

4. If you decided to move more, what form of exercise would you be most likely to choose? When? How much?

5. How do you feel about intentionally speeding up your walking, at least occasionally? On what occasions would this be feasible?

6. If you wanted to regularly interrupt a session when you are sitting down, how would you do it? To what extent would this be feasible?

7. Do you use notifications on your mobile phone? What do you think about receiving notifications from time to time to help you move more, walk faster, and interrupt sessions when you are sitting more often? On what occasions would this be appropriate? How often?
